# Medical Notes and Reflections

**Published:** 1839

**Authors:** 


### Medical Notes and Reflections. By *Henry Holland*, M.D. &c. &c. 8vo. pp. 628. Longmans, 1839.

It would be well for medical science, if some of those who have devoted volumes to fine-spun theories and speculations, had simply recorded the results of their observations and reflections during an active practice of twenty or thirty years. Under this impression we opened the volume before us; and, although we cannot say that we have been entirely free from disappointment on perusing Dr. Holland's book, yet we are sure that it will prove an acceptable present to the profession. The author, during twenty years' practice in this metropolis, has been in the habit of making notes of interesting cases, and of appending to them such reflections as occurred to his mind on these and other occasions. The contents are therefore very miscellaneous, and we shall notice them separately or in classes. Our first paper shall be THERAPEUTICAL, and it will embrace the third, fourth, eighth, ninth, tenth, sixteenth, twentieth, twenty-first, twenty-sixth, thirty-second, and thirty-third Chapters.

### I. Bleeding in Affections of the Brain.

We rejoice in finding the opinion of so experienced a physician as Dr. Holland delivered so strongly against the indiscriminate use of the lancet in these cases. Although much improvement in this point of practice has resulted from the valuable observations of Dr. M. Hall, Dr. Gooch, and others, yet, whether it arise from the difficulties of the diagnosis, from the deception created by the *temporary* benefit so often following, or from the necessity which many medical men seem to consider themselves to be under, of always employing some visibly active proceedure; certain it is, that with the great bulk of the profession, abstraction of blood constitutes not only the chief, but sole means of relieving cerebral affections. In our schools the subject of blood-letting is often treated too carelessly; few teachers dwell sufficiently upon the necessity of care and discrimination in expending the invaluable fluid; while others, indeed, recommend free depletion, without much troubling their hearers with restrictions of any kind. It is too often forgotten, not only that there is a large class of cases in which such proceeding is absolutely injurious and inappropriate; but also, that in the cases in which depletion is allowed by all to be required, it is oftentimes carried to a most improper extent, producing a long train of nervous symptoms, or laying the foundation of subsequent serious chronic disease—sequelæ not always thought of when the case is exhibited to the admiring class, as an example of what (properly termed) *bold* treatment will effect. Thus it happens, that, one of the first errors the young practitioner has to unlearn, often is, a too unlimited confidence in the powers of the lancet. He finds, though too often from some unhappy results, that, care and discrimination are required to prevent its being the instrument of mischief in the place of good. To him works like the present are always useful and acceptable guides.

Many of our readers will readily acknowledge the truth of the following passage.

"Is not depletion by bleeding a practice still too general and indiscriminate in affections of the brain, and especially in the different forms of paralysis? I believe that the soundest medical experience will warrant this opinion. The vague conception that all these disorders depend upon some inflammation or pressure, which it is needful to remove, too much pervades and directs the practice in them: and, if the seizure be one of sudden kind, this method of treatment is often pursued with an urgent and dangerous activity. Little heed is taken of the many cases where the symptoms depend upon irritation alone,—or on loss of nervous power,—or on deficient circulation of the blood within the brain,—or on altered qualities of this blood,—or, it may be, on morbid changes in the nervous substance itself. Theory might suggest that, in some of these various cases, the loss of blood leads to mischief.—Experience undoubtedly proves it; and there is cause to believe that this mischief, though abated of late years, is still neither infrequent nor small in amount.

It is certain indeed that there is a state of brain, best perhaps represented to us in its general effects of diminished nervous power, which tends to produce sometimes spasmodic seizures, sometimes delirious or maniacal affections, sometimes palsy of different parts of the body—these effects being in no wise obviated by depletion, but rather increased by all such means; while they are relieved by remedies which tend to excite the energy of the sensorium, and to augment the general power." 39.

Dr. Holland describes a state of the brain in which depletion, from its debilitating effects on the nervous system, must be employed with especial care. It is nearly allied to the state of deficient or altered nervous energy. Probably, the cerebral mass is affected in an analogous manner (although by causes of slow and gradual operation,) to what it was in the experiments of Sir A. Cooper, who produced paralytic and spasmodic affections, by depriving the brain of blood by the ligature of the two vertebrals. However produced, it may manifest itself in every degree, from mere changes in mind and animal spirits, to the production of convulsion and paralysis. Its nature seems much to resemble the changes produced in the brain by old age. A similar state results from anxiety and distress of mind, as also from overstrained intellectual labour.

He next passes in review some of the symptoms usually deemed as justifying, or calling for the use of the lancet, but which sometimes require very opposite means. Thus *coma*, although usually supposed to be dependent on pressure, to be relieved by bleeding, is often a symptom of extreme debility—sometimes expressly induced by depletion. It is a state more nearly allied in such cases to syncope than to pressure. Dr. H. is certain that he has seen cases where the tendency to coma might give strong presumption of pressure, in which large bleeding has induced paralytic attacks which might otherwise have been spared.

*Vertigo.*—He justly observes there are cases, "and these, as I think, of frequent occurrence, where vertigo is the effect of causes wholly remote from fullness or overaction: being in fact a step towards syncope, and a proof of the exhaustion of the nervous power."

His remarks on bleeding in *Paralysis* are very judicious. Allowing that, where it may be susceptible of relief, by emptying the vessels of the head, it has arisen from extravasation or other cause of pressure ; yet, he observes, it frequently results from an instant shock to a portion of the brain or spinal-marrow ; the shock may be of momentary duration, its effects lasting upon the nervous system, while the physical changes are little known to us ; although it is probable that the nervous substance itself is often previously affected. The practice of bleeding immediately after the stroke of palsy is often reprehensible. "The risk, I believe, will generally be less from waiting a certain time,—to observe the effect of what has occurred upon the circulation, the breathing, and the sensibility,—than from hastily taking away blood, at the moment of a great shock to the brain, and before we can rightly appreciate its consequences. This effect upon the greater functions of life gives us, in fact, the best information we can have in guidance of further practice. But this we forfeit in great part, by the disturbance any large depletion makes in the system, and especially upon the organs upon which these functions depend." Even where the fitness is undoubted of bleeding after one paralytic attack for the prevention of another, Dr. H. prefers, as the safer practice, small and repeated bleedings, to copious immediate depletion.

He thus concludes the subject—

"These remarks, and the cautions they suggest, are familiar to many, and to such needless. But I feel assured, from what I have seen, that they ought to be carried further into general practice. The use of the lancet is easy, and gives a show of activity in the practitioner at moments when there appears peculiar need of this promptitude. Current opinions and prejudices are wholly on the side of bleeding; and the complexity and danger of the cases tend to obscure the results of the treatment pursued. The physician needs all his firmness to decline a practice thus called for; where the event is so doubtful, and where death may be charged upon his presumed feebleness or neglect." 47.

He appends some cursory remarks upon some other points connected with the treatment of head-affections.

The *application of cold to the head:* he cautions us against its indiscriminate use, and careless application ; we know its effects vary according to the quantity and mode of application, and as some head-aches are decidedly aggravated by it, although others are relieved by it, it becomes a question, whether all the other more serious affections of the brain are equally relieved by its use, as are those of an inflammatory character. "Unfortunately these are cases (as in apoplexy, &c.) in which generally patients can render least aid. Sometimes they are manifestly uneasy under the application of cold. Their feeling, whenever it can be ascertained, is better than any other test."

The *raised position of the head* is too often indiscriminately insisted upon, for, although it is frequently a most useful precaution, yet at other times the good which results is no equivalent for the irksomeness it causes; while again, in cases of diminished energy of the brain, it is positively improper. "Headache moreover in some instances is brought on, or much augmented, by the upright posture, and subsides when this is changed. In general we may gather from observation which position of the head is best—by the colour of the face, by the state of the breathing, and by the action of the heart or other functions, if the patient be unable to express his own sensations. Perhaps altogether the respiration is the most certain test we can employ in the absence of the latter."

"*Bleeding by leeches from the hæmorrhoidal vessels* might be much more frequently employed than it is in affections of the brain, as well as in those of the spinal cord. I know no mode in which a given quantity of blood can be removed with equal effect in the cases where this is required."

### II. Observations on Various Medicines.

Under this head we propose to collect some of the very useful observations upon the administration of several medicines, which are contained in different parts of the work.

1. But first, a few extracts from the chapter on *Methods of Prescription*. Dr. Holland justly remarks, that although we have abandoned the complex formulæ of former times, yet "the error is still frequent of aiming at a greater number of objects than can be reached at once, or by any single combination of remedies; thereby forfeiting the good to be derived from simpler and more explicit means. Secondary and subordinate symptoms are apt to usurp a place in prescriptions, due to such only as are essential to the character and course of the malady. And it is not too much to affirm that the judgment of the medical man upon a disorder is often warped by the reflection of his own practice. He is apt to look at the symptoms through his own previous treatment of them, as well as through the false opinions of those around: and the trifling or casual symptom of one day gains undue weight the next, from its needless admission among the objects for which he has prescribed. All this is natural in itself, but undoubtedly a serious error in practice. In no way can it better be obviated than by carefully simplifying the character of prescriptions: and avoiding ambiguity, as far as possible, in the intention we assign to their several parts."

After adverting to the difficulty which exists in estimating the effects of the various elements of a prescription, he adds—"the best general rule seems to be, to define in every case, as far as can be done, the main object of present treatment,—to make this the basis of prescription,—and to admit subordinately and under limitation, all such means as apply to collateral symptoms. This rule ought especially to be kept in mind, when a new medicine, or new applications of an old one, are on trial in our hands. Complexity here, especially that which arises from the admixture of new agents in the same prescription, disguises that which it most imports us to know, and which is always learned with difficulty amid the many conditions tending to obscure the effect. In adding to these the uncertainty of combination, which is itself in most cases an experiment, we wrap one doubt within another, and play false with every just principle of medical research."

Dr. Holland recommends the young practitioner to examine for symptoms by some certain rule or method, as tending to give exactitude to his own ideas of the case, and to his powers of communicating them to others: to avoid being betrayed into hasty diagnosis and prognosis, by which his subsequent practice might become embarrassed or negatived "from the desire to redeem a wrong opinion thus given;" and to remember, that in prescribing for a patient, there are numerous circumstances beyond the mere diet and medicine, which call for the careful physician's attention.

He also considers it as a good rule, in the treatment of disease, to examine how far *external remedies* are fitted to the case, before determining those for internal use: which enables us, not only to consider how far the desirable object of relieving the affection by the former can be realized, but also affords us due time for the attentive consideration of the latter.

2. *Sudorific Medicines.*—Dr. Holland lays down these positions in reference to sudorifics:—

"First, that it is more reasonable, as well as beneficial in practice, to have regard to the changes in the circulation producing diaphoresis than to the action of sweating itself. And, secondly, that the amount of perspiration is rarely a just measure of the good obtained: and, that to make this a primary object is likely to give a wrong and injurious bias to the treatment of disease." Upon the whole we think, that, perhaps, he speaks too disparagingly of this class of remedies, believing diaphoresis, as he does, as indicating rather an effect than a cause of change of the state of the disease. But we fully agree with him, that much of the improved practice of our days consists in having abandoned the heating and stimulating plans of treatment which had for their object to force perspiration.

*Opium as a Diaphoretic.*—"Of the various internal means for obtaining diaphoresis, I believe that opium, in one or other of its forms, is the most uniformly certain and beneficial. Its action, in conformity with the views just stated, may be considered to depend on its powers of allaying inordinate circulation, or other excitement of the system. This opinion is justified, as well by the other effects of opium as by the similar influence of various narcotics on the state of the skin.

I allude more especially to opium as a diaphoretic, because I believe that in our modern practice we too much neglect the various and beneficial resources afforded by this medicine. Its action in this way probably depends chiefly on its power of allaying excitement of the vascular system. Whatever be thought of Lind's recommendation of opium in the hot stage of ague, it is certain that in various states of fever and inflammatory disorder, where preceded by sufficient evacuations, no medicine can be more safely used as a sudorific, or with greater collateral advantages." 61.

3. *On the Use of Diluents.*—These are considered under three points of view. "1st. The mere mechanical effect of a quantity of liquid in diluting and washing away matters, excrementitious or noxious, from the alimentary canal."

Dr. Holland speaks strongly in favour of the free use of *water* to produce this effect, in many states of the alimentary canal; when taken fasting, at an agreeable temperature, he has known it conduce more than any medicine, to the due performance of the functions of the alimentary canal, and especially where its secretions have become vitiated. In cases, in which an habitual excess of acid in the lower bowels is a source of distress, half a drachm of carb. sodæ, taken in a large quantity of water, will greatly relieve—being often much more useful, thus given, than when a smaller quantity of water is employed.

He also suggests, that we should make much more use of the surface of the alimentary canal, as a means of conveying cold, than is customary. "I have seen enough of the benefit from cold liquids freely given in the acute stages of gastric disorders, inflammatory and febrile, with express reference to the point of temperature, to justify a more frequent recourse to it in practice. This is a point where the feelings and desire of the patient may fairly be taken in guidance, and we can rarely go wrong in following them."

"2nd. Their influence in modifying certain morbid conditions of the blood." After reviewing the facts at present known, in reference to the absorption of fluids into the circulation, Dr. H. concludes, that our present knowledge will not justify us in prescribing diluents with the direct intention of altering the qualities or proportions of the blood: but that, whenever fluids are demanded by the sensations of the patient, they may be freely and fearlessly given. "Even in *diabetes*, I have never found any continued good from opposing the inordinate thirst of the patient, nor aggravation of what are the essential symptoms of the disorder, by assenting in full to this desire. The restraint as to liquids, to satisfy the thirst of malignant *cholera*, is still less justified by any reasonable principle or experience in practice. The thirst occurring as an ordinary symptom of all *fever*, is for the most part to be viewed as authority for its gratification. In the greater number of cases, more harm will arise from opposing it, than from any excess in the use of liquids to which it can lead."

"3dly. Their effect upon various functions of secretion and excretion, and especially upon those of the kidneys and skin."

He considers that simple diluents, as water, freely given, form our very best sudorifics, and that in many febrile diseases they should be more freely employed.

Also, in those dropsical affections in which it is the object to restore the due action of the kidneys, diluents are very useful, after the due evacuation of the bowels:—a preliminary essential to the due operation of all diuretics. He attributes to the effects, which as diluents they exert on the functions of the kidney and skin, the beneficial results which so often follow the free employment of the continental mineral waters.

4. *Abuse of Purgative Medicines.*—The author acknowledges there is considerable justice in the criticisms made by our continental brethren upon our employment of these. He denounces the idea, countenanced by too many medical men, that a daily evacuation is to be procured as necessary to health; seeing that the constitutions of individuals vary much in this particular. In *dyspepsia*, a long train of maladies often ensue, as the result of too frequent use of purgatives, increasing the torpor they were intended to relieve, and irritating other parts of the canal.

But while blaming the injudicious use of these medicines, Dr. Holland is a strong advocate for their efficient employment in appropriate cases, in which a degree of vigour "is sanctioned to an extent even beyond that to which it is carried in ordinary practice. For it is not paradoxical to affirm that the practitioner who deals too familiarly with these medicines, in all cases, is less likely to use them boldly and efficiently where the need is greatest. The accustomed habit is carried on to the particular instance, and due discrimination is wanting in both." Again, he thinks that, in the cases in which purgatives are really required, the *dose* is often very injudiciously reduced; and, that a dose capable of speedy and powerful effect, exhibited at suitable intervals, is much preferable to small quantities repeated from day to day.

The cases calling for active purgation, are, especially, affections of the head, and of the organs connected with the portal system, certain dropsies, in which the kidneys refuse their office; or, where the body is disordered by certain morbid matters collected and circulated in the blood, as evidenced by the discharge of dark grumous stools, &c. &c. Although purging should generally be limited to the removal of actual disease, yet it may frequently be employed as a preventive of disorder. Thus, in those cases, in which there is a tendency to form accumulations in the vascular system, and morbid deposits, great advantage will attend free purging at intervals, as may be seen in the gouty temperament.

"Nor can the need be doubted of aiding the natural action of the bowels in many cases where there is habitual costiveness, either from particular temperament or the casual condition of life. But I have already pointed out the great abuse by excess in this part of practice, and the importance of giving more scope than is usually done to the natural powers of the organs in relieving any such casualties. I advert to the subject again, as well to enforce this, as also to suggest the value in these cases of the direct combination of *tonics* with aperients; a form of prescription which might well be brought into more general use. In the greater number of instances of this kind, weakness in the proper action of the bowels is the cause of costiveness; and in seeking to remove the effect by means which act through irritation only, we do but add to the mischief. The tonic, conjoined with the aperient, enforces its action, without weakening the organs. These in fact are the cases in which, if there be no irritation of the mucous membrane, bark will itself often act as a laxative. I have known many instances where calomel, colocynth, and gamboge, in large doses, have had little other effect than that of injurious irritation; but where a few drachms of infusion of senna, with decoction of bark, have been amply sufficient in producing the desired action.

This practice is of more especial value in those languid and strumous habits in which strength and good digestion are so carefully to be maintained; and where an irritation, habitually repeated, often becomes the source of active and permanent disease." 108.

5. *Use of Colchicum in Gout.*—We have not space to pursue the author's disquisition on the nature of gout, but will extract some of his observations upon colchicum. He observes, that it is to have too limited an idea of the powers of colchicum, to suppose it is only capable of relieving the paroxysm. "Its power of removing gouty inflammation from the joints, is subordinate to its action on the matter of gout throughout the system: and it is to the latter we must look for explanation of those effects which may thus be deemed specific in every just sense of the term." He conceives it acts through the medium of the kidneys, and observes, that, although three or four large doses of colchicum may suffice to induce a sufficient change of action in these organs, to check or withdraw the inflammation from any given part, yet that if we suspend its use at this point, "the matter of the disease thus drawn into the circulation, and not provided with any further or sufficient egress, may be presumed ready to show itself in fresh attacks of local inflammation. If, on the other hand, the altered action of the kidneys be sustained by the continued use of the medicine, it may be capable of removing the gouty matter from the system altogether; or that surplus of it at least which becomes a source of active disease." Inattention to this point, Dr. Holland believes to be the cause of the occasional anomalous effects attending the use of colchicum, as also of the suspicion, now held by many, of its relieving the present paroxysm at the expense of a more frequent recurrence. He says, "on my experience, however, I believe this opinion to be justified only where the medicine has been used imperfectly: or without other precautions, which are more or less essential to its success. I can scarcely doubt the expediency of carrying its employment beyond the mere relief to the local inflammation of the disease. The remedy, with due care, may be made preventive as well as curative of gout; and, as far as I can judge, with no less safety to the patient." "There is still a tardiness and timidity in its application beyond the mere fit of the gout, which is not warranted by any ascertained risk."

In reference to the precautions mentioned in the above extract, he observes, we should seldom exhibit colchicum, "without the combination of other means, fitted to act freely on the bowels; and calomel, though not perhaps essential, may for the most part be preferred for this purpose. The action of the remedy thus aided, if not more rapid, is certainly more secure. In sustaining fully all the excretions, (for though that of the kidneys be most important it is not solely effective,) we gain the best guarantee that the morbid matter removed from the joints is carried out of the circulation." Dr. Holland prefers the *acetous extract*. "I have given this, in moderate doses, every night for three or four weeks, without a single manifestation of ill effects. Its combinations with calomel and other purgatives in the outset of an attack of gout,—with morphine or other sedative, and with occasional laxatives, in the progress of the malady,—and with alkalies and stomachic bitters in sequel to it, and for the prevention of its recurrence, furnish us with the greater part of what is needful in this disorder, as far as medicine merely is concerned."

6. *On Mercurial Medicines.*—Dr. Holland considers, that these are too frequently used as mere laxatives; and too sparingly and inefficiently, where relief to certain varieties of inflammation, or the absorption of various morbid effusions and excretions are sought for: in which cases small and scattered doses harass the bowels with fretful and irregular action, without being efficient for relief. He says also, that the admixture of other aperients with *calomel*, often impair its mercurial effects, although such may be proper where we only desire to produce prompt and full discharges from the liver and bowels: but "even in cases of obstruction of the bowels, when there is threatening of topical inflammation, calomel adequately given, without the addition of other laxatives, will often be more effectual for relief than in any combination with them. Its single action is much less irritating to parts thus disposed; while there is generally more distinct and speedy exercise of its specific effect upon membranes already in an inflamed state." He speaks highly in favour of the more extensive use of the *oxymuriate*. "I have seen its influence in augmenting the secretions, procuring the absorption of morbid growths, altering the state of the skin in many cutaneous disorders, and changing the character of morbid actions generally throughout the system, in cases where I believe no other medicine, or combination of medicines, would have had equal effect. Its conjunction with bark, steel, sarsaparilla, &c. afford resources of the greatest value in the treatment of disease. And, though otherwise held by common opinion, I think it on the whole as safe a medicine as calomel in the hands of the practitioner: inasmuch as its distribution can be made as equal and determinate; and its effects, from being given in a state of solution, are less likely to be interrupted by mechanical hindrances in the stomach and bowels."

7. *On the Use of Emetics.*—Our author is of opinion, that practitioners of the present day have in many affections substituted injudiciously the use of purgatives for that of emetics, and that in very many complaints we should make a much freer use of the latter than we do. They are pre-eminently useful in the commencement of fever, in various gastric affections (in which all other treatment is often unavailing until the disordered secretions be thus evacuated), in affections of the liver and portal system:—in cases of slight jaundice, as also of fevers of warm climates, (which have such close relation to the functions of the liver and the circulation through the chylopoietic viscera) they are of primary utility. He has found them of great utility in the epidemic influenzas of late years, while as aids to expectoration generally, they act a most important part.

"The use of this remedy in the disorders of *children* is at present much less general than it ought to be. In very many cases emetics would beneficially supersede that employment of purgatives which often adds to the irritation it professes to remove. In the infantile fever for example, which is a type of various disorders, an occasional dose of ipecacuanha, so as to excite vomiting, (especially where there is much of the cough which attends this complaint, and large secretion from the mucous membranes,) will be found more effectual than any other means. It is to be noted, further, that the action of vomiting is for the most part singularly easy in children; more immediate, and generally less distressing than that of purgative medicines." 298.

8. *On the Use of Opiates.*—Dr. Holland observes, that since the free use of purgatives has become established in this country, opium has been much less employed, and although of late years, by reason of the introduction of the preparations of morphia, it has again been more freely resorted to, yet, he thinks, not to the degree it deserves. Moreover it is very frequently administered in a very inefficient dose.

"Yet medical experience does but follow common observation in recognising the inestimable value of sleep in sickness; of the suspension of pain, and the check to all disordered actions, thereby obtained. For pain and sleeplessness, though strictly but symptoms of other ailments, may often in practice be viewed as disorders in themselves, the removal of which is essential to the success of our general treatment.* How frequently do we see a nervous restlessness come over the patient, the consequence of protracted sickness or other causes,—retarding cure, by preventing the due effect of remedies, and receiving no relief

* "A physician of large practice, whom I knew but in the latter days of his life, acted not unwisely, perhaps warily, in carrying about with him a box of small opium pills; with which he often gave relief, or made preparation for it, while yet in the house of his patient."

itself from the means employed for the disease. In such cases the physician is not to submit himself to names or technicalities. The regular course of treatment must be suspended till the hindrance is removed; and even seeming contradictions to this course may safely be admitted for the attainment of the object. Here opium is the most certain and powerful of the aids we possess; and its use is not to be measured timidly by tables of doses, but by fulfilment of the purpose for which it is given. A repetition of small quantities will often fail, which, concentrated into a single dose, would safely effect all we require." 420.

It is well known also, that opium, when used to relieve some very severe forms of painful diseases, is not followed by the same ill-consequences, which succeed to its use in slighter cases. And, truly does he add, "the injurious effects of this substance as a medicinal agent are altogether overrated; there is reason to say, respecting the general action of opium on the animal economy, that no agent of equal and similar power produces its peculiar effects with less detriment to the constitution; nor is there any which admits of such extension of use, if gradually made, without fatal result."

"The power of this remedy in checking various morbid actions in their earliest stage, even such as have no direct relation to the nervous system, is too much neglected in modern practice. In common *catarrhs*, for instance, 20 or 30 drops of laudanum, or an equivalent dose of some other opiate, given with a warm diluent at bed-time, and followed in the morning by whatever laxative may be required, will often arrest altogether a complaint, which the later use of purgatives, antimonials, and salines would only tardily remove." It is one of our most efficient diaphoretics; the doses of Dover's Powder given, being, however, usually too small.

We have not sufficient space to follow Dr. Holland through some very good observations upon the cautious, yet often useful employment of this remedy in inflammation, in cerebral affections, fever, &c. and we will only extract his opinion on the local use of opium—a mode of treatment coming more and more into vogue.

"The *external* application of opium is not sufficiently brought into use, nor is there a due appreciation of all that may be attained in this way. In many cases of painful affection of particular nerves, I have found great benefit from a strong opiate ointment on a blistered surface; or a little of the muriate of morphia sprinkled over it. In the more complex case of gastrodynia, so trying to the skill of the practitioner, though the cause cannot thus be obviated, yet much relief to suffering may often be given by similar means applied to the epigastrium, or other more especial seat of pain. I have found no remedy more beneficial in those irritable forms of local psoriasis, which are so distressing from their obstinacy, than the application of poultices, prepared with a small proportion of a solution of opium, and continued until the state of the skin is thoroughly changed. In many nervous and spasmodic affections, as in some forms of asthma, when we have reason to believe that the medulla spinalis is the chief source of disorder, the same remedies may be applied along the course of the spine; and often with singular good, when all methods of depletion and counter-irritation have proved utterly unavailing." 431.

9. *On the Use of Digitalis.*—We consider Dr. Holland's observations under this head as very important, being calculated to remove the timidity which often proscribes or unduly limits the use of digitalis. His recommendations do not arise from any theory of the action of this substance, but, are of a practical, or rather of an empirical character;—he feeling himself unable to explain many of the anomalies which are often found to accompany its employment.

"After all that has been written upon this medicine, there is reason to doubt whether its capabilities have yet been brought into full use. An agent which can thus alter the heart's action, and, under management, safely sustain this alteration for a considerable time, is a very important one in the hands of the practitioner; affording means wherewith to control, more or less directly, all the actions of the system. It is, however, this singular sedative effect on the heart and the occasional suddenness of the change so produced, which creates an apprehension as to its use, not warranted by what we learn from exact observation. Though employing the medicine somewhat largely in practice, I do not recollect a case in which I have seen any injurious consequences from this cause;—none such, certainly, as were not speedily relieved by its discontinuance, and other means of easy adoption.

I am far from inculcating neglect of the symptom just mentioned. But while admitting that deviation below the average standard of the pulse is less frequent and considerable than excess above it, and that certain morbid states of the brain are sometimes thus indicated, I feel assured that alarm is too readily taken in the early effects of digitalis on the heart, even should intermittent action be one of these. Similar effects occur habitually from so many other causes affecting the body, without injury which can be especially referred to this symptom, that if there be justification on other grounds for sustaining the action, and care given to watching it, the practice may generally be pursued without any risk to countervail the good." 545.

Not only the combination with digitalis of tonics and stimuli, does not impair its specific effects, but even extends them, particularly, where it is especially desired to act on the kidneys in debilitated habits. "I have frequently given digitalis in the ordinary doses, for weeks together, in combination with the træ. ferri mur. or tinct. ferri am., without finding cause to withdraw or intermit the medicine; and this in cases where the patients were far advanced in years, and the circulation become feeble from disease. It would be difficult to find any single combination more effective than this in old cases of general dropsy: in œdematous swellings from debility; or in the anasarca following scarlet fever, where, together with weakness, there is still left an excited and irritable state of the arterial system. The action of the fox-glove as a diuretic, seems even to be quickened and sustained by being conjoined with the steel; while the latter acts apparently as a safeguard against some of its peculiar effects. It cannot be doubted that this medicine may often be given with safety and advantage, even where the action of the heart is already extremely irregular. The irregularity arises occasionally from causes which the digitalis is capable of lessening or removing:—as from fluid actually effused. But besides this less direct influence, it is probable that its immediate action on the heart, tending under certain circumstances to produce intermittent pulsation, may, under other circumstances correct the irregularity already existing. And accordingly I have often found perfect equality of beat to ensue for a time upon its use, where the motions of the heart had been very unequal in their ordinary state." Dr. Holland has even found it useful where actual structural mischief existed; at least in one form, viz. the enlarged and flaccid heart, connected sometimes with dropsy.

" Respecting the several forms under which digitalis is used, there is some reason to believe the *infusion*, rightly prepared, to be the most certain and beneficial in its effects: and admitting of every combination with other medicines which can in any case be required. This, indeed, is an instance where, from the nature of the agent, it is singularly needful that the quality of its preparations should be equal and uniform. It is certain that many of the inequalities, and seeming anomalies in the effects of digitalis are owing to neglect of this important precaution." 551.

10. *On Antimonial Medicines.*—" I annex this subject to the preceding, having chiefly in view what is still the insufficient use in English practice, of those medicines expressly sedative to the actions of the circulation. I have noticed this in the instance of digitalis; and it is equally true as regards some of the antimonial preparations, and the tartarized antimony especially. The opinions held in relation to the supposed sudorific effect of these medicines, have prevented due attention being given to their influence in allaying inordinate action; either of the brain, as in mania and delirium of different forms: or more generally in inflammation, or febrile states of the whole system." 552.

Of its relation to mercury and bleeding in inflammatory disease, Dr. Holland thus expresses himself.

" As a febrifuge in the most direct sense of the term, it may be doubted whether we possess any more direct or speedy in effect. It is impossible to have witnessed its effects, when adequately given, in acute pneumonia or bronchitis, or croup, without recognizing the same fact, and seeing that where it does not wholly supersede bleeding as a prior remedy, it at least abridges the demand for this; and comes in aid of it, by inducing the same state of the vascular system throughout the body. Its relation to mercury in these cases is that of most interest; the effect apparently the same, so as to make it in many instances doubtful which of the two remedies may be best relied upon for instant use; yet the mode of operation, presumably very different." 554.

As to its dose and mode of administration. " The influence, indeed, of dose and manner of employment, curious and important in all cases, well deserves notice here. Our practice in England (limited, as I have said, by older views,) seldom goes beyond such use of them in inflammatory disorders, as to sustain nausea for a time, and thereby repress the circulation; and even this intention is often made subordinate to the forcing of perspiration, as a means of relief. It is certain that the full value of the medicine is not thus attained: and though we yet want proof of the efficacy or need of the large doses of tartar-emetic employed by some of the Continental physicians, it seems certain that those we use might be beneficially encreased in relation to their sedative effect."

Although he is averse to confusing or negativing the operation of so powerful an agent by complex combinations, he considers that one between tartar-emetic and opium as very excellent, especially, in cases of mania and cerebral irritation, and, more reservedly, and after depletion in purely inflammatory affections, as pneumonia, bronchitis, rheumatism, meningitis, &c.

" Reverting to the use of this combination in affections of the brain, it is remarkable how speedy and assured the effect often is. In the wild restlessness of delirium tremens this is sometimes very strikingly seen. In fevers attended with much cerebral disturbance its employment has been attended with similar good. And in various forms of spasmodic disease I have experience of the same kind; giving evidence of benefits which are not so readily or completely obtained by the use of opium alone." 558.

